# Rapidly adapted community health strategies to prevent treatment interruption and improve COVID-19 detection for Syrian refugees and the host population with hypertension and diabetes in Jordan

**DOI:** 10.1093/inthealth/ihac083

**Published:** 2022-12-28

**Authors:** Ruwan Ratnayake, Fatma Rawashdeh, Raeda AbuAlRub, Nahla Al-Ali, Muhammad Fawad, Mohammad Bani Hani, Saleem Zoubi, Ravi Goyal, Khaldoun Al-Amire, Refqi Mahmoud, Rowaida AlMaaitah, Parveen K Parmar

**Affiliations:** International Rescue Committee, Jordan Office, Amman, 11183, Jordan; Department of Infectious Disease Epidemiology, London School of Hygiene and Tropical Medicine, Keppel Street, London, WC1E 7HT, UK; International Rescue Committee, Jordan Office, Amman, 11183, Jordan; Department of Community and Mental Health Nursing, Jordan University of Science and Technology, Irbid, Ar-Ramtha, 22110, Jordan; Department of Community and Mental Health Nursing, Jordan University of Science and Technology, Irbid, Ar-Ramtha, 22110, Jordan; International Rescue Committee, Jordan Office, Amman, 11183, Jordan; International Rescue Committee, Jordan Office, Amman, 11183, Jordan; International Rescue Committee, Jordan Office, Amman, 11183, Jordan; Division of Infectious Diseases and Global Public Health, University of California San Diego, San Diego, California, 92093, USA; International Rescue Committee, Jordan Office, Amman, 11183, Jordan; Division of Cardiovascular Disease, Jordanian Ministry of Health, Amman, Jordan; Department of Community and Mental Health Nursing, Jordan University of Science and Technology, Irbid, Ar-Ramtha, 22110, Jordan; Keck School of Medicine, University of Southern California, Los Angeles, California, 90033, USA

**Keywords:** cohort analysis, community health workers, COVID-19, diabetes mellitus, displaced persons, health services accessibility, humanitarian assistance, hypertension, Jordan, non-communicable diseases, refugees, Syria

## Abstract

**Background:**

We evaluated community health volunteer (CHV) strategies to prevent non-communicable disease (NCD) care disruption and promote coronavirus disease 2019 (COVID-19) detection among Syrian refugees and vulnerable Jordanians, as the pandemic started.

**Methods:**

Alongside medication delivery, CHVs called patients monthly to assess stockouts and adherence, provide self-management and psychosocial support, and screen and refer for complications and COVID-19 testing. Cohort analysis was undertaken of stockouts, adherence, complications and suspected COVID-19. Multivariable models of disease control assessed predictors and non-inferiority of the strategy pre-/post-initiation. Cost-efficiency and patient/staff interviews assessed implementation.

**Results:**

Overall, 1119 patients were monitored over 8 mo. The mean monthly proportion of stockouts was 4.9%. The monthly proportion non-adherent (past 5/30 d) remained below 5%; 204 (18.1%) patients had complications, with 63 requiring secondary care. Mean systolic blood pressure and random blood glucose remained stable. For hypertensive disease control, age 41–65 y (OR 0.46, 95% CI 0.2 to 0.78) and with diabetes (OR 0.73, 95% CI 0.54 to 0.98) had decreased odds, and with baseline control had increased odds (OR 3.08, 95% CI 2.31 to 4.13). Cumulative suspected COVID-19 incidence (2.3/1000 population) was suggestive of ongoing transmission. While cost-efficient (108 US${\$}$/patient/year), funding secondary care was challenging.

**Conclusions:**

During multiple crises, CHVs prevented care disruption and reinforced COVID-19 detection.

## Introduction

There is emerging recognition of the need for new models for the management of non-communicable diseases (NCDs) in humanitarian settings.^1^ The Syria crisis highlights the burden borne by 6 million refugees displaced regionally.^2^ In northern Jordan, we conducted a population-based survey among Syrian refugees living outside of camps and found that 34/100 adults aged ≥18 y reported a diagnosis of diabetes, hypertension or both conditions.^3^ Refugees access healthcare through clinics supported by non-governmental organizations (NGOs) that provide monthly consultations and medications at no or low cost, and Ministry of Health (MOH), private clinics, secondary care and laboratories that charge fees.^4^ Hence, NCD management for refugees is highly fragmented and dependent on NGO clinics.^4^

There is evidence that primary care integration of community health workers or volunteers (CHWs/CHVs) can improve the continuity of NCD care.^5–8^ In humanitarian settings, community networks are typically used to extend primary care services for maternal, child and reproductive health.^9^ Comparatively, there is little evidence on the effectiveness of CHW/CHV strategies for NCD management.^9^

Since 2011, the International Rescue Committee (IRC) has administered CHV programs for Syrians and vulnerable (uninsured) Jordanians. We originally sought to evaluate the effectiveness of CHVs (via blood pressure and/or glycemic control and outcomes of early disease detection, biological monitoring, adherence to medication and insulin, and screening and referral for acute complications) on NCD disease control outcomes using a stepped wedge trial design (ClinicalTrials.gov, NCT04229667). The trial was initiated in February 2020, just before coronavirus disease 2019 (COVID-19) was detected in Jordan in March.^10^ As NGO clinics were required to close, the IRC rapidly adapted the trial's CHV intervention toward a simplified and telephone-administered version and expanded it to cover clinic cohorts. This drew on ad hoc strategies to support continuity of care for HIV/AIDS and diabetes patients during conflict in the Central African Republic, Yemen and Mali.^11,12^

This study aimed to evaluate the CHV intervention within the clinic catchment area of two IRC-supported clinics in the cities of Ramtha (Irbid Governorate) and Mafraq (Mafraq Governorate) in northern Jordan that supported 1300 Syrian refugees and vulnerable and uninsured Jordanian patients with hypertension and/or diabetes. We evaluated this program using a cohort study, a qualitative study and a cost-efficiency study. The primary objective was to evaluate trends in the utilization and potential effects on stockouts, medication adherence, new complications and completed referrals. The secondary objectives included investigation of changes in disease control outcomes (blood pressure and glycemic control) pre-/post-strategy initiation, disease control predictors and implementation success and costs. Other secondary objectives included estimating the incidence of suspected COVID-19 in households, as a measure of case detection sensitivity, and calculating the proportion of patients completing testing, as a proxy for access to public health systems.

## Materials and Methods

### Study design, setting and participants

A prospective cohort study of the clinic cohorts of hypertension and diabetes patients aged ≥18 y was conducted. Monthly trends in programmatic outcomes and suspected COVID-19 detection were assessed. Changes in disease control outcomes and predictors of disease control were evaluated. A qualitative study and cost-efficiency analysis were conducted to evaluate implementation and cost per patient of the CHV program. The setting was the IRC clinic catchment areas in Mafraq Qasabah District (Mafraq Governorate, 31 079 refugee population) and Ramtha District (Irbid Governorate, 28 976 refugee population) on the Syrian border. The clinics were closed from 21 March to 17 November 2020.

The clinic cohorts of hypertension and diabetes patients (N=1300) were eligible for inclusion for the intervention and study. The study’s inclusion criteria were previously diagnosed patients aged ≥18 y with hypertension, diabetes or both conditions and vulnerability (most vulnerable Syrians classified as per the United Nations Higher Commissioner for Refugees [UNHCR] Vulnerability Assessment Framework, uninsured and vulnerable Jordanians, with physical disabilities or other vulnerability as identified by staff). A nurse reviewed the electronic health information system (HIS) records to establish which patients were active/inactive, hospitalized and pregnant. While patients were continually included in the program, those starting by 30 September 2020 were enrolled in the study to give ≥5 mo study time.

### Intervention

**Figure 1.**
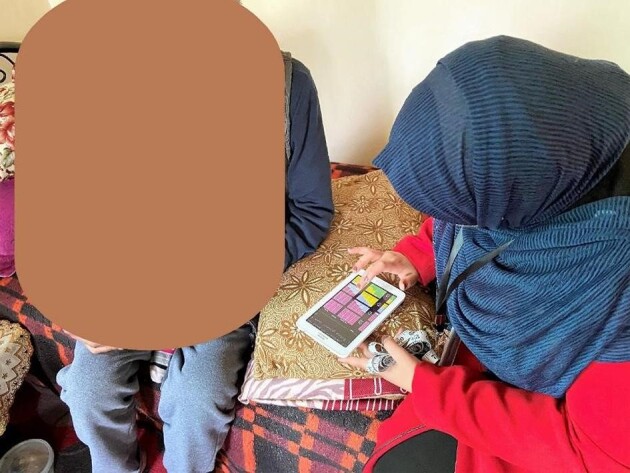
Depiction of a monthly home visit (in February 2020, before COVID-19) with a CHV, where CommCare is used for patient monitoring (here, with cardiovascular disease risk charts) and height, weight, blood pressure and random blood sugar are taken.

The original home visit intervention was based on a literature review, prevalence survey^3^ and a participatory workshop with patients, CHVs and partners (Figure 1).^13^ The remote strategy was refined by clinicians and the research team to fill gaps. It focused on mitigation of secondary impact on NCD outcomes due to the disruption to clinical care^12^ and infection prevention, surveillance and linkage to testing for COVID-19, as summarized using a predefined framework for primary care of NCDs (Table 1).^14^ Formal counseling on diet and lifestyle was deemed too time-intensive, and biological monitoring was not possible without a household visit. For clinical questions, CHVs linked patients with clinic staff.

**Table 1.** Interventions and domains based on the Kane framework^14^ and covered by the remote CHV intervention

Domain	Remotely delivered interventions	Guidelines and/or tools used
1. Case finding	Not delivered	–
2. Modify risk factors	• Monthly education on self-management of diabetes and hypertension, including WhatsApp messaging • Secondary prevention using self-management protocols, identification of danger signs, early detection of complications, discussion of cardiovascular risk	• CommCare patient file^20^ • WHO Total CVD Risk charts • Health education aids for patients and by condition on self-management, dosing and medication, family support, danger signs^15,18^
3. Essential medicines	• Medications and/or insulin delivered monthly to a local pharmacy • Monthly check for patient stock of medication and link to the pharmacy if there is a potential stockout	• IRC clinic telehealth service and linkage with local pharmacies
4. Essential diagnostics	No diagnosis was done as CHVs are not medical professionals	
5. Systematic monitoring and evaluation	• CHVs used CommCare and tablet computers to systematically record patient information • Patient information was used to monitor the program in real time through supervision tasks and tracking of metrics	• Monitoring using Power BI dashboard to track metrics • Monthly supervisor meeting for training and improvement of quality of care using data from CommCare
6. Decentralized care, task-shifting and multidisciplinary clinical care	• Provide linkage with primary care for treatment and medication adjustment • Provide self-management support • Provide basic psychosocial support	• Existing models from low- and middle-income countries^5-8^ • Simplified motivational interviewing^15^ • IFRC psychosocial tool^15^
7. Standardized treatment and diagnosis	• Monthly screening to assess the availability of medications and medication adherence	• Algorithms for telephone-based foot screening and detection of complications^18^ • CASE adherence questionnaires^6,19^
8. Standardized referral pathway and follow-up appointments	• Screen for acute complications of hypertension and diabetes, psychosocial problems, with a referral for secondary or psychiatric care as needed	• Verification system including validation of request for referral by supervisor (a nurse) • Ad-hoc referral system for acute complications (Tekafel, Caritas, MOH, etc.) • Referral system to IRC psychosocial care and psychiatric services
9. COVID-19 prevention, screening and referral to testing	• Screen for COVID-19 compatible symptoms with referral to testing and emergency care, if needed • Infection prevention using WhatsApp-delivered messages and a discussion of key preventative messages relating to shielding inside the household, personal hygiene, avoidance of gatherings and early detection of infection through testing	• COVID-19 screening and referral algorithm based on the community case definition^16^ • Visual messages delivered through WhatsApp that used validated Arabic infection prevention messages^17^ (see Figure 2)

Abbreviations: CASE, Center for Adherence Support Evaluation (CASE) Adherence Index; CVD, cardiovascular disease; IRC, International Rescue Committee; MOH, Ministry of Health.

**Figure 2.**
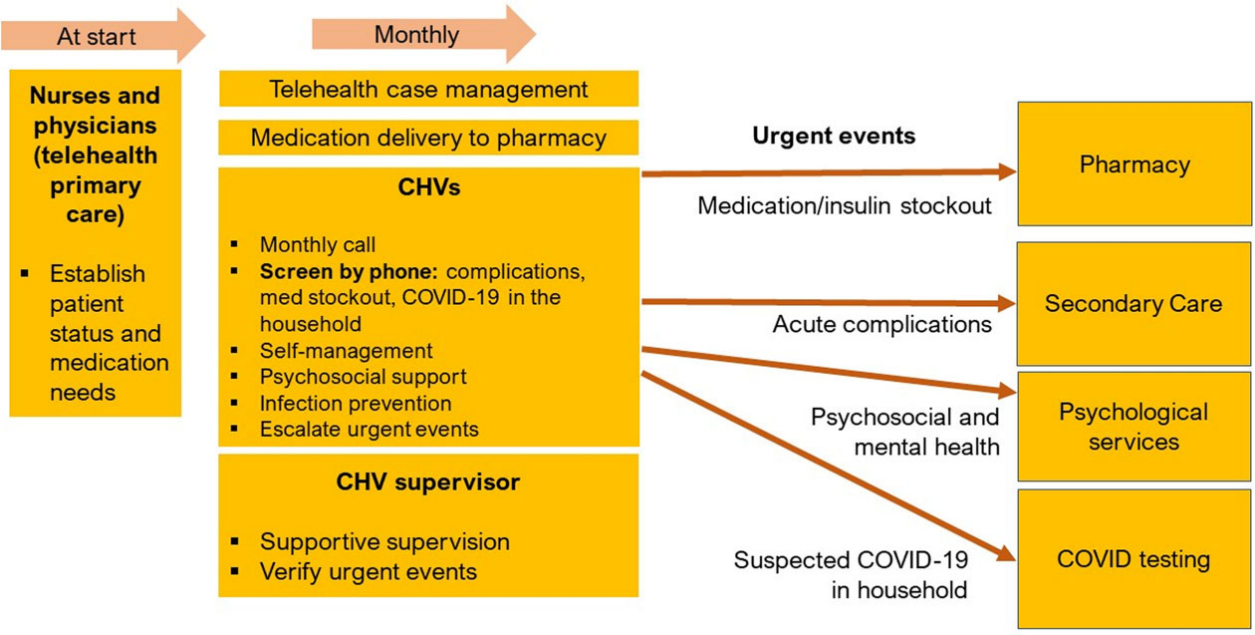
Operational strategy for the CHV program. CHV, community health volunteer.

**Figure 3.**
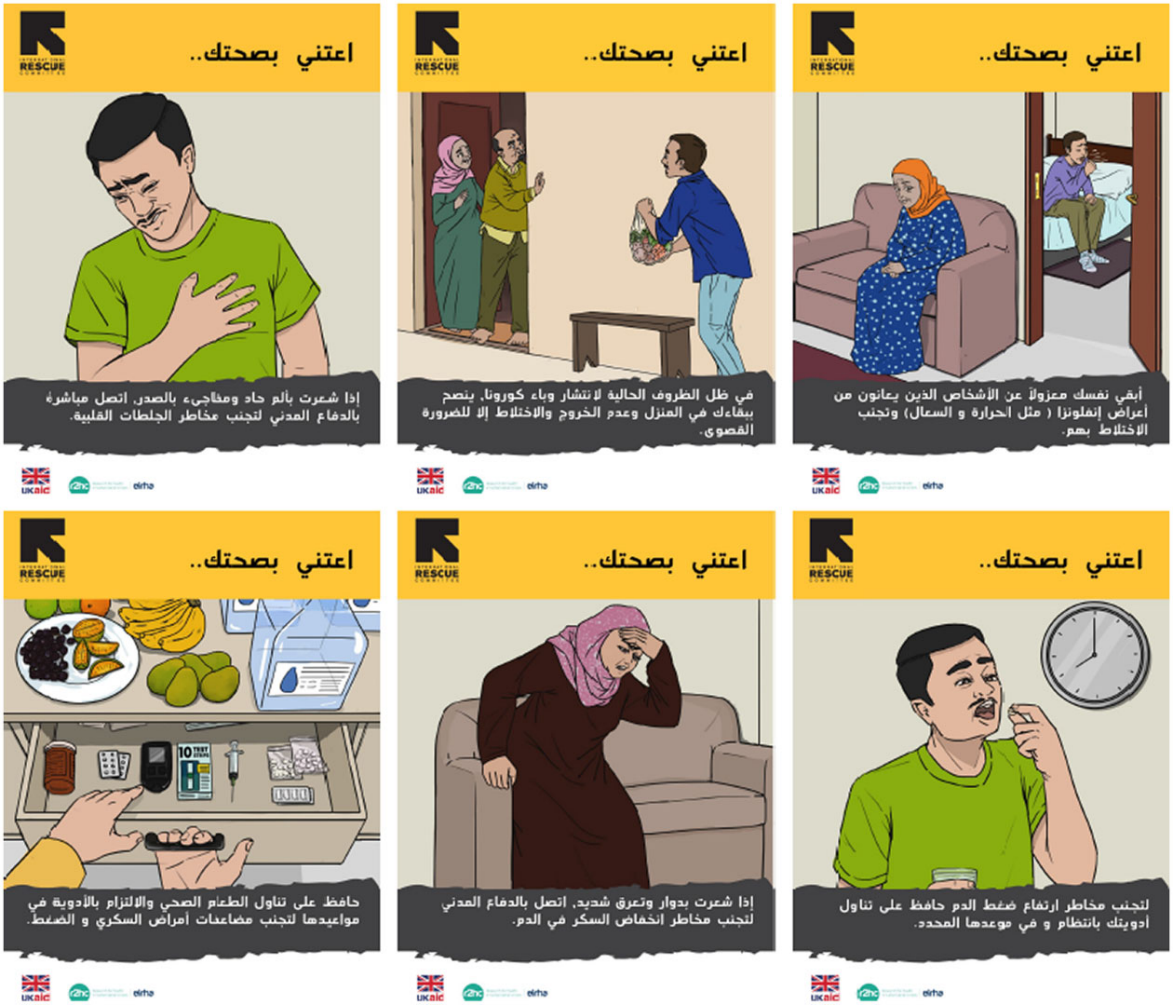
COVID-19 and non-communicable disease management messages delivered by WhatsApp. Designed by Add Value Creators (see Acknowledgements).

The operational strategy is outlined in Figure 2. Nurses first called the patient to establish stability and arrange for monthly medication delivery at a local pharmacy. CHVs then took consent to participate in the intervention and study and document risk factors and preconditions. Monthly, CHVs used a CommCare tablet application to provide a telephone consultation to enquire about medication stockouts, identify new complications, provide psychosocial support and coach on self-management of disease and COVID-19 infection prevention.^15^ When new complications or psychiatric needs were identified, supervisors referred patients to the IRC's psychosocial team and other service providers (e.g. emergency, specialists, surgery, psychiatry). CHVs conducted symptom screening for COVID-19 for patients and household members during the past 2 wk and severe symptoms during the past 3 d (to match national test-eligibility criteria) (see Supporting Text 1 for case definitions).^16^ When a suspected case required testing, supervisors rescreened and referred to a testing center. CHVs followed all referrals and testing to monitor completion and outcome. After the monthly call, CHVs sent, via WhatsApp, a set of messages on NCD self-management and infection prevention (Figure 3).^17^ To promote patient retention, CHVs obtained a second telephone number of another family member (or close contact) during the initial visit, to be contacted if the patient missed a monthly telephone call.

### Data collection and analysis

The HIS was used to retrieve demographic and clinical data. Other variables were sourced through the consultation (i.e. pregnancy status, smoking status, comorbidities, complications related to an NCD and vulnerability markers [registration status, reliance on cash assistance]).

Programmatic outcomes included the monthly proportion completing a call, reporting a medication/insulin stockout, acute complication (i.e. shortness of breath, chest pain, swelling in legs, new numbness/weakness in extremities, sudden headache, sores that do not heal and/or new loss of vision) and requiring referral to secondary care.^18^ The total number of referrals for psychosocial, psychiatric and emergency care were tallied. Adherence during the past 5/30 d was defined as the proportion reporting that they had taken medications for 5/the past 5 d and 28/the past 30 d.^6,19^

Disease control was defined as blood pressure (BP)<140/90 mmHg (hypertensives) and random blood glucose (RBG)≤200 mg/dl [10.1 mmol/L] (diabetics). Systolic BP (SBP), diastolic BP (DBP) and RBG were extracted from the HIS before clinic closure (last readings in February/March 2020) and after clinic reopening (first readings in November 2020). The final outcome was alive and retained, defaulted (no completed call during the last 3 mo), excluded (became pregnant, hospitalized or receiving care elsewhere) or dead.^20^

Suspected COVID-19 incidence was assessed as the number of suspected cases meeting the community case definition^16^ among patients (i.e. fever and cough or fever and shortness of breath in the past 3 d or contact with a laboratory-confirmed case in the past 2 wk) detected divided by the number of patients X 1000 population. Confirmed COVID-19 cases tested positive by PCR.

### Data analysis

Analyses were restricted to individuals with complete data, for a particular analysis. Monthly counts of consultations and proportions of programmatic outcomes and optimal adherence at 5/30 d were calculated with monthly patients as the denominator. The point estimates and their 95% CIs were graphed. Locally estimated scatterplot smoothing (LOESS) curves were plotted to aid in the visualization of time trends. Secular trends were assessed using a negative-binomial regression model for each proportion as the outcome variable and the month as a predictor variable. To demonstrate non-inferiority of the CHV intervention (i.e. that patients did not do worse after implementation of the program), statistical comparisons of the means of SBP and RBG before and after initiation of the CHV program were conducted using a paired Wilcoxon signed rank sum test. The proportion with disease control pre-/post-initiation of the strategy were compared using χ^2^ tests (or Fisher's exact tests). SBP and BP control analyses and RBG and RBG control analyses included patients with hypertension (and both conditions) and diabetes (and both conditions), respectively.

Predictors of disease control were evaluated using multivariable models. Logistic multivariable models were adjusted for endline disease control (to mark the ultimate outcome of disease control). Linear multivariable models were adjusted for the % change in disease control between baseline and endline (to mark patients who improved or worsened) and for the total number of calls received (to mark intervention uptake). The final model used demographics, baseline status (existing complication, comorbidity status, baseline disease control) and program effects (calls completed, defaulter status, ≥1 medication/insulin stockout, ≥1 referral to secondary care) as potential predictors. Akaike information criteria were used for model selection. Adjusted *R*^2^ or the likelihood ratio test was used to evaluate model fit.

### Qualitative substudy

In-depth interviews (IDIs) and focus group discussions (FGDs) were conducted to explore program implementation from the perspectives of patients, CHVs, supervisors and staff at midline (November 2020) and endline (March 2021). Respondents were purposively sampled by role. IDIs and FGDs were conducted in Arabic and English, using Zoom and in person (Supporting Text 2, question guide). Content analysis included notes the research team took during monthly CHV and clinic meetings.^21^ Quantitative data and qualitative data were interpreted concurrently by RR, PP and FR using a convergent parallel design to integrate findings across methods.

### Costing substudy

Costing data included direct costs (i.e. stipends, medications, equipment), start-up activities (i.e. training, technical support, travel) and indirect shared costs (administration). Using Dioptra software (IRC, New York, NY, USA), the total costs were proportioned according to program use and divided by the total number of patients as the output.^22^

## Results

Following program development/approval, training, piloting and enrollment (March–June 2020), data collection took place from 27 June 2020 to 28 February 2021 (8 mo). Overall, 1119 patients consented by the study cutoff date. At study end, 973 (87.0%) were retained, 110 (9.8%) defaulted, 23 (2.1%) moved out of the catchment area and 14 (1.3%) died. Of the 14 deaths, investigations by clinicians (PP, FR and SZ) determined that no deaths resulted from the CHVs’ action/inaction; 3/14 who died had a PCR-confirmed COVID-19 result (of six tests known to have been conducted). The IRB review of the 14 investigation reports led to recommendations to continue the study without changes to the protocol.

Patients were distributed between Mafraq (53.7%) and Ramtha (46.3%) clinics; 601 (53.7%) had both conditions, 423 (37.8%) had only hypertension and 95 (8.5%) had only diabetes. Of the 696 diabetics, 136 (19.5%) were insulin-dependent. Table 2 describes the baseline characteristics. The majority were female (62.6%), aged 41–65 y (68.0%) and Syrian (88.8%). Almost 40% had an existing comorbidity and nearly all (96.2%) relied on medication from the clinic. Among Syrians, 93.1% were dependent on cash assistance. Nearly half of males (204/418, 48.8%) were smokers.

**Table 2.** Descriptive epidemiology of the cohort by demographics, risk factors, primary NCD diagnoses, comorbidities and existing complications at baseline (N=1119)

Variable	n	N	n (%)
Governorate Mafraq Ramtha	601 518	1119 1119	53.7 46.3
Age group (y) 18–40 41–65 66–80 ≥80	81 761 238 39	1119 1119 1119 1119	7.2 68.0 21.3 3.5
Gender Male Female	418 701	1119 1119	37.4 62.6
Nationality Syrian Jordanian	994 125	1119 1119	88.8 11.2
Primary income (main categories only) UNHCR cash assistance Daily labor or paid work Cash transfers Loans/borrowed money	548 172 129 118	1119 1119 1119 1119	49.0 15.4 11.5 10.5
Registration (Syrian only) With UNHCR With Jordan Ministry of Interior	985 929	994 994	99.1 93.5
Reliant on UNHCR cash assistance (Syrian only)	929	994	93.1
Health status at baseline			
Current smoker Male Female	305 204 101	1119 418 701	27.3 48.8 14.4
Primary diagnoses Hypertension only Diabetes only Insulin-dependent Hypertension and diabetes Insulin-dependent	423 95 27 601 109	1119 1119 95 1119 601	37.8 8.5 28.4 53.7 18.1
Requires medication for NCD	1076	1119	96.2
Any comorbidity^1^	444	1119	39.7
Asthma	78	1119	7.0
COPD	82	1119	7.3
Cardiovascular disease (CVD, CVA, IHD)	395	1119	35.3
Kidney disease	68	1119	6.1
Existing complications Peripheral neuropathy Sores that will not heal Amputation due to disease	275 34 18	1119 1119 1119	24.6 3.0 1.6

Abbreviations: COPD, chronic obstructive pulmonary disease; CVA, cerebrovascular accident; CVD, cardiovascular disease; HIS, health information system; IHD, ischemic heart disease; NCD, non-communicable disease; UNHCR, United Nations Higher Commissioner for Refugees.

1 Including preregistered comorbidities in the HIS, including asthma, CVD, CVA, IHD, COPD and/or kidney disease.

### Programmatic outcomes

**Figure 4.**
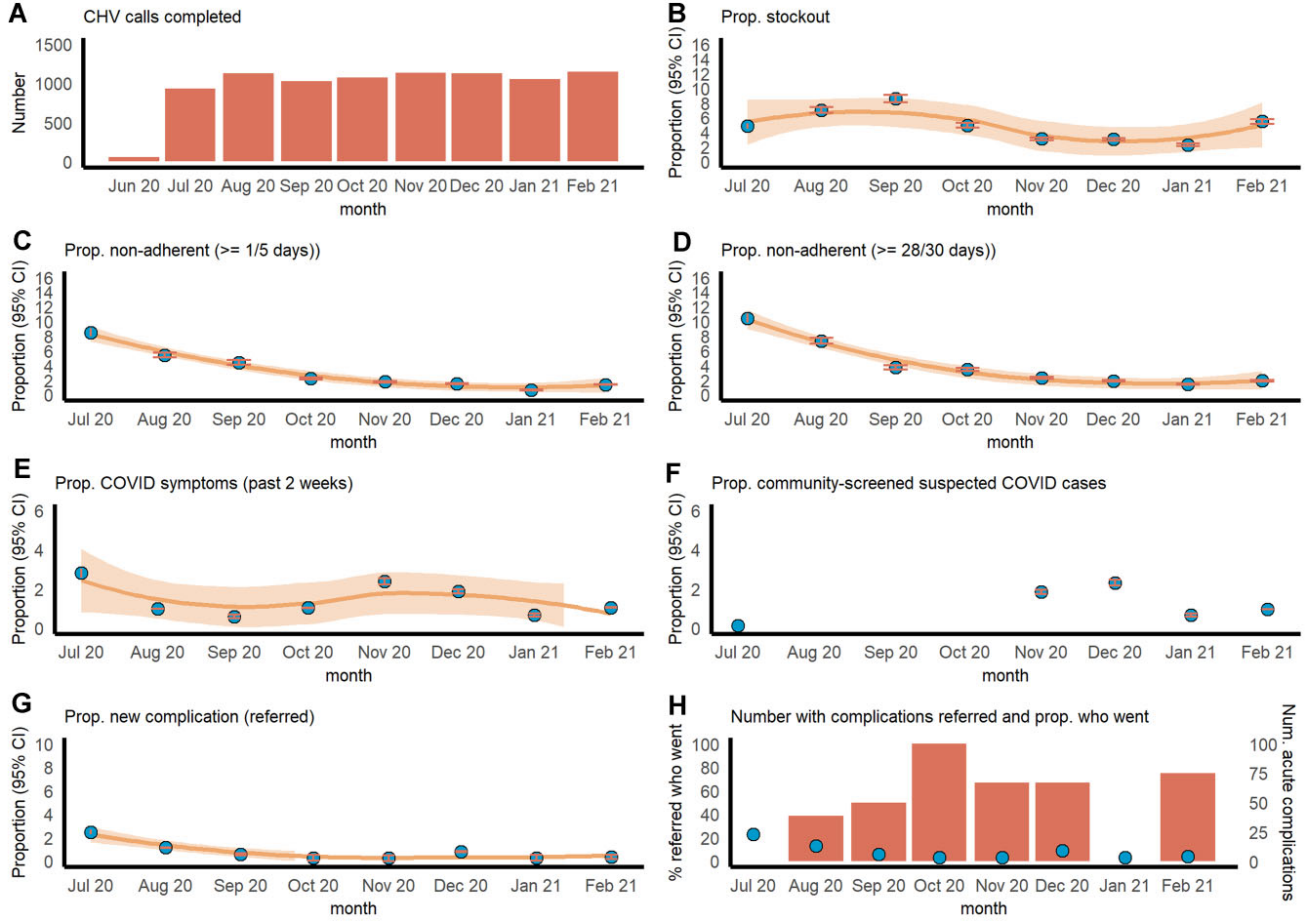
Time trends in programmatic outcomes (blue dots) and their 95% CIs (orange shading) and LOESS regression line (orange line and shading). Note the difference in scales between measures. LOESS, locally estimated scatterplot smoothing.

The mean monthly number of consultations was 961 (range 58–1144); the lower end (58 patients) was registered during 27–30 June 2020 (Figure 4A). Consultations remained stable with no significant change (p=0.12). The mean monthly proportion with medication or insulin stockouts was 4.9% (range 2.3%–8.6%) (Figure 4B). While there were no significant changes over time, stockouts were highest in September 2020 where there were external constraints in medication supply. The mean monthly proportion of non-adherence remained below 5% of all patients: 3.2% (range 0.7%–8.5%, 5-d recall) and 4.1% (range 1.4%–10.4%, 30-d recall) (Figure 4C,D). The mean monthly decrease in non-adherence was 1% (p=0.00).

The mean monthly proportion reporting symptoms compatible with COVID-19 disease in the past 2 wk was 1.4% (∼16 patients, range 0.6%–2.8%) (Figure 4E). No suspected cases were identified from August to October 2020. The mean monthly increase in suspected COVID-19 cases referred to testing was 1% (p=0.04), coinciding with a national increase in infections in late 2020 (Figure. 4F). The mean proportion with acute complications referred to secondary care was 2.4% (∼27 patients, range 0.4%–8.8%), with a mean monthly decrease of 1% (p=0.00) (Figure 4G). The mean proportion of referrals completed among patients with acute complications was 55.6% (∼six patients) but dropped to zero and was 100% in some points, with no significant change in level (Figure 4H).

### Disease control, acute complications and COVID-19 disease

Acute complications were detected among 204 (18.1%) patients and included swelling in legs (n=62, 30.4%), numbness in extremities (n=45, 22.1%), sudden headache (n=41, 20.1%), sores that do not heal (n=37, 18.1%), chest pain (n=34, 16.7%), shortness of breath (n=31, 15.2%) and vision loss (n=18, 8.8%). After supervisor screening, most patients (69.1%) were asked to self-monitor for worsening. Emergent cases (n=63, 30.9%) were referred to secondary care at NGO (n=28, 44.4%), MOH (n=12, 19%) and private clinics (n=23, 36.5%). Thirty-two (50.8%) of 63 referred patients completed the referral.

The cumulative incidence of suspected COVID-19 cases detected by CHVs was 2.3/1000 population. Using a recall of the past 2 wk, 124 (11%) reported symptoms compatible with COVID-19 disease and 23 (2%) had contact with a confirmed case. During the past 3 d, 25 (2.2%) reported symptoms conferring eligibility for testing (Table 3). Fifty-five (4.9%) reported having household members with COVID-19–like symptoms in the past 2 wk with 11 (1.0%) reporting testing-compatible symptoms in the past 3 d. CHVs flagged 51 (4.5%) patients or household members as suspected cases and immediately discussed how to undertake infection prevention measures for household members. Supervisors verified that 18 (35.3%) of the 51 suspected cases met testing criteria and referred patients to a testing center. Ten (55.6%) of the 18 cases tested positive for severe acute respiratory syndrome coronavirus 2 (SARS-CoV-2).

**Table 3.** COVID-19 outcomes for patients and patient households (N=1119, unless otherwise stated).

COVID-19 outcomes	n	n/N (%)
Patient		
COVID-19 symptoms in patient (during past 2 wk)	124	11.1
Fever plus cough or shortness of breath (during past 3 d)	25/124	20.2
Had contact with a confirmed case (during past 2 wk)	23	2.1
Patient-household member		
COVID-19 symptoms in household member (during past 2 wk)	54	4.8
Fever plus cough or shortness of breath (during past 3 d)	11/55	20.0
Had contact with a confirmed case	13	1.2
Suspected COVID-19 case (CHV referred to supervisor)	51	4.6
Verified and referred to testing	18/51	35.3
Tested positive by PCR	10/18	55.6

SBP, RBG and disease control measures were compared pre-/post-initiation of the CHV program (Table 4). The majority (84.9%) with hypertension had endline SBP values available, while fewer with diabetes (44.2%) had endline RBG values available. The null hypothesis of no difference between SBP and RBG remained true as mean values for SBP among patients with hypertension (136 vs 134 mmHg) and RBG among patients with diabetes (251 vs 260 mmol/dl) did not change between time periods. The null hypothesis of no difference between the disease control measures for SBP (<140 mmHg) and RBG (<200 mmol/dl) was met as there was no apparent worsening in control between time periods (except for disease control [SBP] for patients with both conditions).

**Table 4.** Comparison of SBP, RBG and disease control by condition for patients who returned to the clinic once it opened

		Preclinic closure		Postclinic opening	
Variable	Proportion of patients with both baseline and endline values	mean	Range	mean	range
SBP					
Hypertension	359/423 (84.9)	136	98–200	134	87–183
Both conditions	475/601 (79.0)	137	97–205	138	95–218
RBG					
Diabetes	42/95 (44.2)	251	100–450	260	127–497
Both conditions	292/601 (48.6)	222	81–533	224	35–587
		n/N (%)		n/N (%)	
Blood pressure control					
Hypertension	359/423 (84.9)	183/359 (51.0)		202/359 (56.3)***	
Both conditions	475/601 (79.0)	245/475 (51.6)		230/475 (48.4)***	
Glycemic control					
Diabetes	42/95 (44.2)	11/42 (26.2)		13/42 (31.0)	
Both conditions	292/601 (48.6)	139/292 (47.6)		148/292 (50.7)***	

Abbreviations: RBG, random blood glucose; SBP, systolic blood pressure.

Comparison made by paired Wilcox signed rank sum test for continuous variables (SBP, RBG) and Pearson's χ^2^ test for proportions (DC) and Fisher's Exact Test (for anticipated cell sizes <5). Endline values that resulted in a significant test value are in bold and otherwise indicate no change: ***<0.001; **<0.01; *<0.05.

In a logistic regression for endline BP control in patients with hypertension, older age groups (41–65 y, OR 0.46, 95% CI 0.2 to 0.78; 66–79 y, OR 0.54, 95% CI 0.26 to 1.1; and ≥80 y, OR 0.44, 95% CI 0.15 to 1.31) had up to 0.46 times lower odds of having endline BP control compared with the 18–40 y age group (Table 5). Having comorbid diabetes resulted in 0.73 times lower odds of endline BP control (OR 0.73, 95% CI 0.54 to 0.98). Starting with baseline BP control resulted in 3.1 times the odds of endline BP control (OR 3.08, 95% 2.31 to 4.13). In the logistic regression for endline RBG control in patients with diabetes, those affiliated with the Ramtha clinic had nearly two times the odds of endline RBG control compared with patients with diabetes attending the Mafraq clinic (OR 1.76, 95% CI 1.04 to 3.00). Achieving baseline RBG control incurred 3.4 times the odds of endline RBG control (OR 3.44, 95% CI 2.15 to 5.59). Logistic regression models aligned closely with the linear regression models for change in SBP and RBG (see the last two columns of Table 5).

**Table 5.** Multivariate prediction of endline blood pressure control (hypertensives), glycemic control (diabetics), systolic blood pressure (hypertensives) and random blood glucose (diabetics) using potential demographics and baseline health and program indicators

	Logistic regression (OR, 95% CI)		Linear regression (β, 95% CI)	
Demographics	BP control (%)	RBG control (%)	SBP (mmHg)	RBG (mmol/dL)
	Hypertension Both conditions (with endline SBP)	Diabetes Both conditions (with endline RBG)	Hypertension Both conditions (with endline SBP)	Diabetes Both conditions (with endline RBG)
Gender (male)	0.84 (0.62–1.13)	0.76 (0.46–1.25)	−0.78 (−3.24–1.69)	13.38 (−7.02–33.76)
Age group (y) 18–40 41–65 66–79 ≥80	ref 0.40 (0.20–0.78)** 0.54 (0.26**–**1.10) 0.44 (0.15**–**1.31)	ref 1.87 (0.62**–**6.45) 1.38 (0.40**–**5.32) 0.86 (0.03**–**13.74)	ref 6.71 (1.55–11.87)* 5.89 (0.26–11.51)* 8.47 (−0.22**–**17.17)	ref −9.95 (−53.49**–**33.59) −8.75 (−58.17**–**40.67) 6.73 (−103.99**–**117.44)
Nationality (Syrian, Jordanian)	1.09 (0.54**–**2.20)	2.94 (0.90**–**10.46)	−0.60 (−6.38**–**5.18)	−12.33 (−60.69**–**36.04)
Dependence on cash assistance	1.43 (0.79**–**2.61)	0.51 (0.16**–**1.49)	−2.78 (−7.67**–**2.12)	5.17 (−38.07**–**48.40)
Clinic (Ramtha, Mafraq)	0.99 (0.71**–**1.37)	1.76 (1.04–3.00)*	0.44 (−2.25**–**3.13)	−41.57 (−63.47– −19.68)***
Baseline health indicators				
Existing complication (any)	1.26 (0.93**–**1.71)	1.10 (0.67**–**1.81)	−1.69 (−4.19**–**0.80)	1.07 (−19.49**–**21.63)
Diabetes status (for hypertensives)	0.73 (0.54–0.98)*	–	3.44 (0.998–5.88)**	–
Hypertension status (for diabetics)	–	1.80 (0.82**–**4.09)	–	−21.91 (−53.49**–**9.68)
Disease control at baseline	3.08 (2.31–4.13)***	3.44 (2.15–5.59)***	−8.37 (−10.76– −5.98)***	−61.28 (−81.28– −41.28)***
Program indicators				
Total consultations completed	1.05 (0.90**–**1.22)	1.00 (0.76**–**1.33)	0.35 (−0.91**–**1.61)	−6.40 (−17.74**–** 4.94)
Defaulter status	1.14 (0.18**–**6.25)	2.69 (0.17**–**80.2)	−1.69 (−15.81**–**4.7)	−56.88 (−168.96**–**55.19)
Drug stockout (≥1)	0.82 (0.45**–**1.50)	0.81 (0.29**–**2.14)	−0.21 (−5.11**–**4.70)	−1.86 (−38.87**–**42.60)
Referral (≥1)	0.50 (0.67**–**4.92)	1.07 (0.38**–**2.92)	4.56 (−1.65**–**10.77)	−17.98 (−59.54**–**23.58)
Interactions				
Clinic*Drug stockout (≥1)	1.79 (0.76**–**4.92)	1.17 (0.19**–**6.82)	−3.82 (−11.91**–**4.28)	−9.71 (−81.93**–**62.50)
Model fit				
p (likelihood ratio test) and comparison of Adj. R^2^ (full and AIC-reduced models)	**No significant difference (use full)**	**No significant difference (use full)**	**Adj. R^2^=0.07 (full)** Adj. R^2^=0.07 (reduced)	**Adj. R^2^=0.15 (full)** Adj. R^2^=0.17 (reduced)
Variables in AIC-reduced model	Age group Cash assistance Diabetes status Control at baseline Existing complication Referral (≥1)	Nationality Clinic HTN status Control at baseline	Age group Cash assistance Diabetes status Control at baseline	Clinic HTN status Control at baseline

Abbreviations: AIC, Akaike information criteria; BP, blood pressure; HTN, hypertension; RBG, random blood glucose; SBP, systolic blood pressure.

Significant values in bold: ***<0.001; **<0.01; *<0.05.

### Costing study of implementation cost-efficiency

The cost of the program was 108 US${\$}$/patient/year; 83% of costs were from salaries. This would increase to 116 US${\$}$/patient/year if the program was implemented using household visits (i.e. adding costs of blood pressure cuffs, scales, tape measures, glucose machines and strips, sharps boxes and gloves).

### Qualitative study of program implementation

At midline, nine IDIs were conducted with supervisors, clinicians and patients, and three FGDs were conducted with clinicians and CHVs. At endline, four FGDs were conducted with CHVs, supervisors, clinic managers, clinicians and patients. Two major challenges were identified among stakeholders. Communication between program stakeholders was challenging. It was voiced that having all stakeholders (clinics, CHVs, pharmacies) under one management structure would be optimal. In parallel with quantitative findings of ∼50% completed referrals, the expense of referrals and laboratory studies, specialty services remained a barrier for patients who often opted not to avoid care.

*In the beginning, coordination was not easy, and communication was not clear, as there were challenges with two-way feedback [between CHVs and clinic staff]. Everything became more coordinated once we created the WhatsApp group and online tracking sheets* (Clinic manager, quote 1).

*One of [the] hypertensive and disabled patients was screened by the CHVs for complications and referred to me. I referred her to the emergency department as she had symptoms of shortness of breath and chest pain. However, she refused to go because of cost. We convinced her to go urgently, and we will communicate with UNHCR to cover the cost. She stayed in the hospital one day when she went, and she needed admission, but no one agreed to cover the cost. She was advised to pay then seek reimbursement. [As] she had no money, she left against medical advice. It is challenging that there are no [cost] free places for refugees to seek care when referred* (Clinician, quote 2).

Several successes were also identified. Detection of COVID-19 symptoms and processes for referral to secondary care improved over time as CHVs became more comfortable with screening criteria and referral mechanisms. Some patients perceived consistent contact as being critical for maintaining medication adherence, understanding self-management of their disease and preventing infection (quote 3). Patients felt supported during a difficult time, as many were isolated and had limited means of receiving critical health education and support; this enabled psychological care seeking (quotes 4 and 5). Several instances were mentioned by patients where CHVs successfully coordinated care between pharmacies and clinics to prevent stockouts.

*At first, I was careless, not interested in what the CHV was telling me, every time they told me about signs of an emergency complication of my disease. Then one day, I got those symptoms and remembered what the CHV told me. Alhamdulillah, they rescued me from confirmed death. Since that day, I started believing in those CHVs and how much they care about me and have helped me* (Patient, quote 3).

*[Regular calls] give us the feeling that we are important, and people care about our health* (Patient, quote 4).

*The CHV provided me with psychological support and linked me with a counselor, motivated me to care about my health, and told me that my life is a priority* (Patient, quote 5).

## Discussion

There is an emerging body of evidence on simplified models for chronic disease care disruption due to acute conflict, disasters, and now, a pandemic.^11,12^ In 2019, IRC clinics had prioritized a cohort of Syrian and vulnerable Jordanian hypertension and diabetes patients at high risk of developing severe disease and reliant on free medications/insulin. CHVs ensured continuity of care by providing an alert and response system for acute complications and referral, suspected COVID-19 disease, and testing, and continuously monitoring patients. Although conducted over a short period, the strategy suggests benefit for continuity of care and filling gaps in the non-clinical components that are difficult to deliver in a busy clinic. Patient and providers perspectives highlighted that continuity was possible, with quantitative results showing high uptake, attendance and adherence. Alerts to stockouts, complications, psychosocial and referrals, and COVID-19 suspected cases were routinely detected and manageable. Disease control showed non-inferiority in that patients did not have worsening disease control while being maintained by the CHVs, without direct care. The intervention was cost-efficient (108 US${\$}$/patient/year/International Dollars (INT)${\$}$218/patient/year), comparable with that of a single multidisciplinary consultation administered by Médecins Sans Frontières in Irbid (clinic visit, education, medications/insulin, psychosocial care: INT${\$}$209–253).^23^

CHVs detected an incidence of suspected COVID-19 cases of 2.3/1000 population, similar to the 2.1/1000 population detected by CHVs from March to April 2020 during an early, low-incidence phase in Thailand^24^ and reflective of epidemic peaks in late 2020 in Jordan. Half of the patients completing testing were positive. The yield is notable as linking refugees to testing had proved challenging due to distrust, stigma and poor availability.^25^ COVID-19 surveillance by CHWs has also been done at scale; the Accredited Social Health Activism program in Bihar, India, rapidly trained 15 000 CHWs in surveillance and early response.^26^

Our study has important limitations. The choice of study design was restricted to a single-group pretest–post-test design that could be implemented with the emergency intervention. The lack of randomization, a control group and monthly biological measurement precludes attribution of the impact to the intervention. The use of multivariate statistics for predictors of disease outcome helps to elucidate potential further avenues for research. In-person counseling, psychosocial support, biological monitoring and visual inspection of complications require evaluation for feasibility, quality of care and safety, as has been done for the management of malnutrition in humanitarian settings.^27^ It is difficult to generalize study findings to settings where home visits are possible. A duration of 8 mo is insufficient to observe long-term changes in behaviors, biological outcomes and mortality. The short duration of the CHV intervention was due to the anticipated transition from its role as the bridge to primary care to the eventual reopening of primary care clinics and increasingly limited humanitarian funding. As the reopened clinics caught up with patient volume, the endline RBG measures were incomplete and mixed RBG, fasting blood glucose and HbA1c measurements. This introduces bias towards patients who received RBG measures. It would have been preferable to perform all qualitative research in person vs through Zoom. While this was not possible, we do not feel the differences in the results between approaches are appreciably different as the same systems for consent, building rapport and interview guidance were present. However, carrying out focus groups would have been more seamless in person and better for establishing rapport among participants.^28^

Several lessons for CHW/CHV programming for NCDs were learned. First, communication systems are critical. NCD management is complex, and the CHV program therefore enabled responsive, real-time, clinical and program feedback, monthly discussions on programmatic indicators, all via data collection using CommCare and an internal WhatsApp platform that linked CHVs, supervisors and clinics in a supervision and feedback loop. Second, the outcomes demonstrated that patient needs were not excessive and could be managed through primary care. Medication and insulin stockouts were consistently low due to the “push” delivery to pharmacies and proactive monitoring by CHVs. CHVs were able to engage patients regarding the need for psychosocial support, emphasizing the importance among crisis-affected populations of offering mental health and psychosocial awareness and referral alongside NCD care, where services exist.^29^ The level and referral acute complications resulted in a number of alerts that were practicable to NGO-supported or private clinics. The availability of non-urgent, low-cost/free secondary care (e.g. foot clinics, ophthalmology, etc.) remained scarce, making referral an ad hoc process. Where free care could not be assured, patients often declined to seek care due to costs. This lack of a secure essential referral pathway for refugees has been discussed extensively with only a slight improvement in terms of funding, referral options and clinical eligibility.^30^ Alternatives have been proposed including training for clinic staff on secondary care for common complications^1^^3^ and supplementing with conditional cash transfers to broaden the choice of expenditures.^31^

Finally, the data shed light on which patients are most vulnerable and have the most to gain from CHVs via predictors for poor endline disease control measures. These included those aged >40 y, with both conditions and poor baseline control. A similar risk group of older patients with poor disease control fared worse on disease control in a longitudinal cohort receiving primary care in Irbid.^32^ For stable patients, CHVs could instead provide foundational self-management protocols on a less frequent basis, which remains a major gap for displaced populations.^33^

## Conclusions

Amidst the severe disruption brought on by COVID-19, a rapidly adapted community health strategy addressed the challenge of continuous management of NCDs among refugees and vulnerable Jordanians and offered a transformative opportunity to expand how CHVs can support primary care systems and address social and equity issues for refugees. These community linkages are key for bridging access to healthcare and knowledge among refugees and vulnerable Jordanians but cannot alone address structural barriers, namely the lack of referral options. There remains a need to evaluate the effectiveness of integrated CHW/CHV and primary care management with home visits in humanitarian settings, and its feasibility, quality, safety of care and cost-effectiveness. We intend to modify the original cluster randomized trial design to carry out such an evaluation in the future.

## Supplementary Material

ihac083_Supplemental_Files

## Data Availability

Individual-level patient data cannot be shared publicly because they pertain to a vulnerable population in a humanitarian setting and contain potentially identifying and sensitive information. These data are available from the International Rescue Committee's Institutional Review Board (IRB) subject to a data sharing agreement. Deidentified monthly aggregate data used for the trend analyses are provided in the Supplementary Files. Qualitative data will be shared on reasonable request after signing the data access and use agreement and vetting and provision of approval by the IRC IRB. Requests may be addressed to the corresponding author or humansubjects@rescue.org.

## References

[1] Slama S , Kim HJ, Roglic G et al. Care of non-communicable diseases in emergencies. Lancet. 2017;389(10066):326–30. 27637675 10.1016/S0140-6736(16)31404-0

[2] UNHCR . Syria Regional Refugee Response: Total Registered Syrian Refugees. Available at: https://data2.unhcr.org/en/situations/syria [accessed 22 December 2022].

[3] Ratnayake R , Rawashdeh F, AbuAlRub R et al. Access to care and prevalence of hypertension and diabetes among Syrian refugees in Northern Jordan. JAMA Network Open. 2020;3(10):e2021678. 33052405 10.1001/jamanetworkopen.2020.21678 PMC7557515

[4] Mouawad E , Adler A, International Rescue Committee. Equitable access to health services: lessons for integrating displaced populations into national health systems. New York, NY, USA: International Rescue Committee; 2021.

[5] Neupane D , McLachlan CS, Mishra SR et al. Effectiveness of a lifestyle intervention led by female community health volunteers versus usual care in blood pressure reduction (COBIN): an open-label, cluster-randomised trial. Lancet Glob Health. 2018;6(1):e66–73. 29241617 10.1016/S2214-109X(17)30411-4

[6] Newman PM , Franke MF, Arrieta J et al. Community health workers improve disease control and medication adherence among patients with diabetes and/or hypertension in Chiapas, Mexico: an observational stepped-wedge study. BMJ Glob Health. 2018;3(1):e000566. 10.1136/bmjgh-2017-000566 PMC5841495 29527344

[7] Jafar TH , Gandhi M, de Silva HA et al. A community-based intervention for managing hypertension in rural South Asia. N Engl J Med. 2020;382(8):717–26. 32074419 10.1056/NEJMoa1911965

[8] Peiris D , Praveen D, Mogulluru K et al. SMARThealth India: A stepped-wedge, cluster randomised controlled trial of a community health worker managed mobile health intervention for people assessed at high cardiovascular disease risk in rural India. PLoS One. 2019;14(3):e0213708. 30913216 10.1371/journal.pone.0213708 PMC6435227

[9] Miller NP , Ardestani FB, Dini HS et al. Community health workers in humanitarian settings: scoping review. J Glob Health. 2020;10(2):020602. 33312508 10.7189/jogh.10.020602 PMC7719274

[10] Yusef D , Hayajneh W, Awad S et al. Large outbreak of coronavirus disease among wedding attendees, Jordan. Emerg Infect Dis. 2020;26(9):2165–7. 32433907 10.3201/eid2609.201469 PMC7454095

[11] Besancon S , Fall IS, Dore M et al. Diabetes in an emergency context: the Malian case study. Confl Health. 2015;9:15. 25937831 10.1186/s13031-015-0042-9 PMC4416388

[12] Ferreyra C , O'Brien D, Alonso B et al. Provision and continuation of antiretroviral therapy during acute conflict: the experience of MSF in Central African Republic and Yemen. Confl Health. 2018;12:30. 29988565 10.1186/s13031-018-0161-1 PMC6027556

[13] Parmar PK , Rawashdah F, Al-Ali N et al. Integrating community health volunteers into non-communicable disease management among Syrian refugees in Jordan: a causal loop analysis. BMJ Open. 2021;11(4):e045455. 10.1136/bmjopen-2020-045455 PMC8061821 33879489

[14] Kane J , Landes M, Carroll C et al. A systematic review of primary care models for non-communicable disease interventions in Sub-Saharan Africa. BMC Fam Pract. 2017;18(1):46. 28330453 10.1186/s12875-017-0613-5 PMC5363051

[15] International Federation of Red Cross and Red Crescent Societies (IFRC), International Federation of Pharmaceutical Manufacturers & Associations (IFPMA). Healthy lifestyle toolkit. Geneva, Switzerland: International Federation of Red Cross and Red Crescent Societies (IFRC); 2016.

[16] Jordanian Ministry of Health; Diagnostic and treatment protocol for patients with novel coronavirus (COVID-19). Amman, Jordan: National Committee for Epidemiology, Jordanian Ministry of Health; 2020.

[17] Syria Research Group (SyRG) , Alhaffar M. COVID messages for Syrian communities - Arabic. Available at: https://www.lshtm.ac.uk/sites/default/files/2020-04/SyRG-COVIDmessages_for_Syrian_communities.pdf [accessed 22 December 2022].

[18] International Rescue Committee. Package of essential non-communicable diseases interventions for humanitarian settings (PEN-H). New York, NY, USA: International Rescue Committee; 2020.

[19] Mannheimer SB , Mukherjee R, Hirschhorn LR et al. The CASE adherence index: a novel method for measuring adherence to antiretroviral therapy. AIDS Care. 2006;18(7):853–61. 16971298 10.1080/09540120500465160 PMC2567829

[20] Khader A , Farajallah L, Shahin Y et al. Cohort monitoring of persons with diabetes mellitus in a primary healthcare clinic for Palestine refugees in Jordan. Trop Med Int Health. 2012;17(12):1569–76. 23051859 10.1111/j.1365-3156.2012.03097.x

[21] Elo S , Kyngä H. The qualitative content analysis process. J Adv Nurs. 2008;62(1):107–15. 18352969 10.1111/j.1365-2648.2007.04569.x

[22] International Rescue Committee. Dioptra. International Rescue Committee (IRC); 2021. Available at: https://www.dioptratool.org/ [accessed 22 December 2022].

[23] Ansbro E , Garry S, Karir V et al. Delivering a primary-level non-communicable disease programme for Syrian refugees and the host population in Jordan: a descriptive costing study. Health Policy Plan. 2020;35(8):931–40. 32621490 10.1093/heapol/czaa050 PMC8312704

[24] Kaweenuttayanon N , Pattanarattanamolee R, Sorncha N et al. Community surveillance of COVID-19 by village health volunteers, Thailand. Bull World Health Organ. 2021;99(5):393–7. 33958828 10.2471/BLT.20.274308 PMC8061662

[25] Singh NS , Abrahim O, Altare C et al. COVID-19 in humanitarian settings: documenting and sharing context-specific programmatic experiences. Confl Health. 2020;14(1):79. 33292392 10.1186/s13031-020-00321-w PMC7676860

[26] Singh S , Bahadur Singh L. Training community health workers for the COVID-19 response, India. Bull World Health Organ. 2022;100(02):108–14. 35125535 10.2471/BLT.21.286902 PMC8795851

[27] Van Boetzelaer E , Zhou A, Tesfai C et al. Performance of low-literate community health workers treating severe acute malnutrition in South Sudan. Matern Child Nutr. 2019;15(Suppl 1):e12716. 30748111 10.1111/mcn.12716 PMC7199048

[28] Renosa MDC , Mwamba C, Meghani A et al. Selfie consents, remote rapport, and Zoom debriefings: collecting qualitative data amid a pandemic in four resource-constrained settings. BMJ Glob Health. 2021;6(1):e004193. 10.1136/bmjgh-2020-004193 PMC7798410 33419929

[29] Gyawali B , Harasym MC, Hassan S et al. Not an ‘either/or’: integrating mental health and psychosocial support within non-communicable disease prevention and care in humanitarian response. J Glob Health 2021;11:03119. 34804510 10.7189/jogh.11.03119 PMC8590828

[30] Akik C , Ghattas H, Mesmar S et al. Host country responses to non-communicable diseases amongst Syrian refugees: a review. Confl Health. 2019;13:8. 30949232 10.1186/s13031-019-0192-2 PMC6431037

[31] Lyles BE , Chua S, Barham Y et al. Improving diabetes control for Syrian refugees in Jordan: a longitudinal cohort study comparing the effects of cash transfers and health education interventions. Confl Health. 2021;15(1):41. 34034780 10.1186/s13031-021-00380-7 PMC8145855

[32] Ansbro E , Homan T, Prieto Merino D et al. Clinical outcomes in a primary-level non-communicable disease programme for Syrian refugees and the host population in Jordan: a cohort analysis using routine data. PLoS Med. 2021;18(1):e1003279. 33428612 10.1371/journal.pmed.1003279 PMC7799772

[33] Elliott JA , Das D, Cavailler P et al. A cross-sectional assessment of diabetes self-management, education and support needs of Syrian refugee patients living with diabetes in Bekaa Valley Lebanon. Confl Health. 2018;12:40. 30214472 10.1186/s13031-018-0174-9 PMC6134700

