# Supplementary material for: Rapidly adapted community health strategies to prevent treatment interruption and improve COVID-19 detection for Syrian refugees and the host population with hypertension and diabetes in Jordan
Source: Int Health. 2022 Dec 28;15(6):664–75. doi: 10.1093/inthealth/ihac083 (PMC10629964; doi:10.1093/inthealth/ihac083)
Supplement: ihac083_Supplemental_Files [file ihac083_supplemental_files.zip › S1_text.docx]

**Supplementary Text 1: Case definitions for COVID-19, Jordan**

Taken from: Diagnostic and treatment protocol for patients with novel coronavirus (COVID-19) issued by the Jordanian Ministry of Health and approved by the National Committee for Epidemiology (updated Saturday, October 31, 2020)^[1]^

1. **Confirmed case**

It is a case that is laboratory proven by a PCR examination through a positive result for the detection of SARS-CoV2 virus.

1. **Suspected case**

It is the case in which the owner complains of

- A high temperature of more than 37.5 º or more with a cough.
- The presence of 3 or more of the following combined symptoms (cough, cold, sore throat, muscle pain with or without joint pain, unexplained shortness of breath, loss of sense of taste or smell, or both).
- A high temperature with a sore throat, with a loss of sense of taste, smell, or both.
- A case that meets the definition of acute and severe respiratory infections or their symptoms similar to influenza.

1. **Possible case**

- A- The patient whose owner complains of symptoms of respiratory infection (fever, cough, etc.) and has been in contact with a confirmed or probable case, or the person has been epidemiologically associated with cases in a focus in which at least one confirmed case has been diagnosed.
- B- A suspected case, according to what was mentioned in the above definition, and the radiograph showed changes consistent with Covid-19 disease, as follows:
  - A chest radiograph showing circular hazy opacities spreading to the sides and down the lung.
  - A stratified image of the chest showing multiple opacities, often circular in shape, in both lungs, resembling opaque glass, and spreading to the sides and bottom of the lung.
- C- The condition in which the owner recently started to complain of symptoms of high temperature, with loss of the sense of smell or taste, or both, in the absence of any other reason.
- D- A person who died without knowing the cause of death and was suffering from difficulty breathing before his death and was in contact with a confirmed or probable case or epidemiological related to a focus in which at least one of the confirmed cases.

1. **Contacted case**

- A person who was exposed to any of the following during the two days preceding the onset of respiratory symptoms and extending to the fourteenth day from the onset of symptoms of a confirmed or probable case:
  - Face-to-face contact with a confirmed or probable case over a distance of 1 m or less for at least 15 minutes.
  - Physical and direct contact with a confirmed or probable case.
  - If a medical personnel takes direct care of a confirmed or probable case without using personal protection means.

1. **Definition of death from Covid-19 disease**

Death resulting from a disease that is clinically consistent with a probable or confirmed case of Covid-19 disease, provided that there is no other clear cause of death that is not related to Covid-19 disease such as road accidents, and here it should not There is a complete recovery period between illness and death.

References

1. Jordanian Ministry of Health. Diagnostic and treatment protocol for patients with novel coronavirus (COVID-19). Amman, Jordan: National Committee for Epidemiology, Jordanian Ministry of Health, 2020 October 31, 2020. Report No.
