# Supplementary material for: Rapidly adapted community health strategies to prevent treatment interruption and improve COVID-19 detection for Syrian refugees and the host population with hypertension and diabetes in Jordan
Source: Int Health. 2022 Dec 28;15(6):664–75. doi: 10.1093/inthealth/ihac083 (PMC10629964; doi:10.1093/inthealth/ihac083)
Supplement: ihac083_Supplemental_Files [file ihac083_supplemental_files.zip › S2_text.docx]

**Supplementary Text 2: Guiding questions for qualitative research**

1. Interview guide (community health volunteers, clinicians, and supervisors)

Tell me a positive story (yours or a colleagues) about a patient served by the CHV program, involving the CHV intervention specifically.

If you have one, please tell me a negative story (yours or a colleagues) about a patient served by the CHV program, involving the CHV intervention specifically.

Are you involved in managing the CHVs? What is your role?

What are positive and negative aspects of the CHV intervention?

How does the CHV program help or hinder clinical care in this remote delivery context?

What aspects of your job are working well with regards to this program?

What are the most challenging parts of your job with regards to this program?

How well are you able to coordinate/not coordinate well with relevant stakeholders: the CHVs, RNs, MDs, patients, supervisors, etc.?

How does the policy both in IRC and nationally affect your work, if at all?

What, if anything, would you change about this program

Is there anything else you would like to share?

1. Interview guide (patients)

Tell me about your experience with the CHV/NCD remote health intervention where nurses and CHVs contacted you and your medications were delivered to a local pharmacy.

In which ways was this program helpful to you? What did your CHV do that helped you directly, and that the nurse could not do?

What did your CHV that did not help? Don’t worry, we will never share your answers. What would be useful for a CHV to do outside of the clinic in the future?

What, if anything, would you like to learn more about from your CHV?

What, if anything, would you change about this program?

Is there anything else you would like to share?

What, if anything, would you definitely want to see continue in this program?

What did you think about remote service delivery by clinics and CHVs?

Please tell me 3 things you learned from your CHV.

How did your CHVs help during COVID, if at all?
